# Integrated Strategy Improves the Prediction Accuracy of miRNA in Large Dataset

**DOI:** 10.1371/journal.pone.0168392

**Published:** 2016-12-21

**Authors:** Bin Xue, David Lipps, Sree Devineni

**Affiliations:** Department of Cell Biology, Microbiology and Molecular Biology, School of Natural Sciences and Mathematics, College of Arts and Sciences, University of South Florida, Tampa, Florida, United States of America; University of Texas at San Antonio, UNITED STATES

## Abstract

MiRNAs are short non-coding RNAs of about 22 nucleotides, which play critical roles in gene expression regulation. The biogenesis of miRNAs is largely determined by the sequence and structural features of their parental RNA molecules. Based on these features, multiple computational tools have been developed to predict if RNA transcripts contain miRNAs or not. Although being very successful, these predictors started to face multiple challenges in recent years. Many predictors were optimized using datasets of hundreds of miRNA samples. The sizes of these datasets are much smaller than the number of known miRNAs. Consequently, the prediction accuracy of these predictors in large dataset becomes unknown and needs to be re-tested. In addition, many predictors were optimized for either high sensitivity or high specificity. These optimization strategies may bring in serious limitations in applications. Moreover, to meet continuously raised expectations on these computational tools, improving the prediction accuracy becomes extremely important. In this study, a meta-predictor mirMeta was developed by integrating a set of non-linear transformations with meta-strategy. More specifically, the outputs of five individual predictors were first preprocessed using non-linear transformations, and then fed into an artificial neural network to make the meta-prediction. The prediction accuracy of meta-predictor was validated using both multi-fold cross-validation and independent dataset. The final accuracy of meta-predictor in newly-designed large dataset is improved by 7% to 93%. The meta-predictor is also proved to be less dependent on datasets, as well as has refined balance between sensitivity and specificity. This study has two folds of importance: First, it shows that the combination of non-linear transformations and artificial neural networks improves the prediction accuracy of individual predictors. Second, a new miRNA predictor with significantly improved prediction accuracy is developed for the community for identifying novel miRNAs and the complete set of miRNAs. **Source code is available at:** https://github.com/xueLab/mirMeta

## Introduction

MicroRNAs (miRNAs) are short non-coding RNA (ncRNA) sequences that are produced mainly through a two-step proteolytic cleavage from primary miRNAs (pri-miRNAs) [1], which are transcribed from genome at many types of locations, including genetic loci, intron regions, intergenic regions, etc. Pri-miRNAs have normally several hundreds of nucleotides and each forms a structure composed of several stem-loop motifs. One of the stem-loop motifs, which should have about 70 nucleotides, is cleaved inside nucleus by RNase-III enzyme Drosha [2]. The product has a two-nucleotide overhang at the 3’-end and is called precursor miRNA (pre-miRNA). Pre-miRNA is then transported into cytoplasma and is further cleaved by RNase-III protein Dicer to remove the loop region [3]. The final product is a miRNA:miRNA duplex about 22 nucleotides long. One of the strands in the duplex is called guide strand and is able to form base-pairing with mRNA in the presence of RNA-Induced Silencing Complex (RISC) [4] to regulate the translation of that mRNA by blocking the translation or by inducing mRNA degradation [5].

MiRNAs form the largest family of gene expression regulators [6]. In human genome, about 2000 miRNAs were reported [7] and these miRNAs were estimated to regulate ~60% of human genes [8]. Clearly, characterizing the specific regulation between miRNAs and mRNAs is critical for deciphering various gene expression profiles. The very first step for characterizing the regulation of mRNA by miRNAs is to identify all the miRNAs in a genome. This is not a trivial task. Although a fair amount of miRNAs have been observed, it is still largely unknown if or not novel miRNAs could be found. This question becomes even more complicated when taking into consideration that individual genomes may have significant amount of sequence variations, such as SNPs [9, 10] and other mutations [11]. Many of these sequence variations in humans are disease specific [12–16] and may have significant roles in the regulation of disease associated genes [17–20]. However, it is not completely clear if or not and how these sequence variations may impact the biogenesis of miRNAs. From a mechanistic point of view, these sequence variations may have profound influence on the transcription of DNA sequences, the structure of RNA transcripts, and the interaction between RNA transcripts and other molecules. Consequently, these sequence variations may influence the biogenesis and function of miRNAs [21, 22]. Therefore, to characterize the subtle influences of RNA sequences on the biogenesis of miRNAs, high-accuracy predictor is required. Nonetheless, developing high-accuracy methods to predict miRNAs based on nucleotide sequence is still an open challenge in computational RNA biology.

To predict miRNAs from large scale transcriptomic data, many computational tools have been developed using different strategies. The first group of methods were built on sequence homology and/or conserved stem-loop structures, such as miRscan [23], MiRseeker [24], miRAlign [25], microHARVESTER [26], miREval [27]. The second group of methods introduced various modifications or added additional filters to the methods in the first group. Examples include: integrating sequence profile into homology search [28]; PalGrade combining thermostability analysis [6]; RNAmico applying Support Vector Machine (SVM) as a secondary structure filter [29]; miRPred using linear genetic programming [30]. Methods in the third group applied various machine learning techniques on sequential and/or structural features to predict miRNAs. This group of methods normally achieve higher prediction accuracy. Multiple machine learning techniques have been used to build predictors of this group, such as support vector machine used by triplet-SVM [31], MiRFinder (SVM) [32], and miRPara [33]; Bayes networks adopted by BayesMiRNAFind [34]; random forest in MiPred [35]; and probability model employed by ProMiR [36]. In all these computational tools, parameters of the tools need to be optimized in pre-selected datasets. A popular dataset used by many predictors contained hundreds of samples [31–38]. However, since the actual number of human miRNAs in current version of miRBase is reaching ~2000 [7], the relatively small size of the afore-mentioned dataset may be a concern for the application of those predictors. It is unclear if the predictors that have been developed using small training datasets are able to perform similarly well on large dataset or not. This question is becoming critical in the big-data era. In addition, many predictors may not have the preferred prediction accuracy that is high enough for direct applications. Therefore, how to further improve the prediction accuracy becomes another challenge.

Recently, the authors improved the prediction accuracy of protein intrinsic disorder using meta-strategy [39, 40]. Meta-strategy (or consensus strategy) is a powerful strategy to improve prediction accuracy by integrating different individual predictors. Very often, individual predictors are built using different techniques, input features, infrastructures, and datasets. Consequently, the true positive, false positive, true negative and false negative predictions of individual predictors are very different. Therefore, integrating these individual predictors is able to combine their true predictions and to improve the final prediction accuracy [41]. The meta-strategy has also been successfully applied in many other areas, such as protein fold recognition [42], protein secondary structure prediction [43, 44], protein interaction [45, 46], protein subcellular locations [47, 48], post-translational modification [49], promoter prediction [50], nucleosome organization [51], and mass-spectrometry analysis [52]. In addition to meta-strategy, the authors also demonstrated that preprocessing of input data by shifting angles improved the prediction accuracy of protein dihedral angles significantly [53]. The angle-shift transformation changed the distribution of samples in the one-dimensional angular space and therefore improved the prediction accuracy significantly. This angle-shift technique is now a standard method in protein dihedral angle prediction [54]. Both of the meta-strategy and the preprocessing of input features provide nonlinear transformation of the distribution of samples in the phase space. In terms of non-linear transformation, meta-strategy and preprocessing of input data are essentially the same as various deep learning techniques [55, 56] that are becoming more and more appealing in the field of machine leaning.

## Methods

### Meta strategy

**Fig 1:**
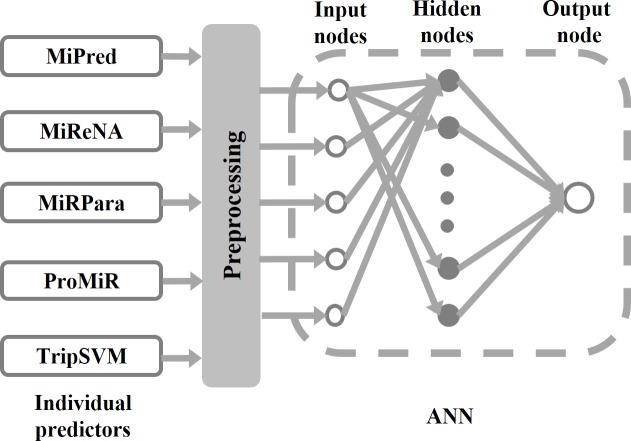
Infrastructure of the meta-predictor. Query sequence is input into each individual predictor. The outputs of individual predictors are preprocessed and then fed into an ANN to make a new prediction, which is the output of meta-predictor. Therefore, the meta-predictor is composed of individual predictors, preprocessing modules, and ANN. The parameters of ANN will be trained using datasets containing both positive and negative samples of miRNAs. Although five individual predictors were shown in the figure, the meta-predictor could be made from any number of individual predictors out of five. The total number of possible meta-predictors is 26. The final meta-predictor mirMeta contains all five individual predictors.

Different predictors may have different true predictions in the same set of samples. Therefore, integrating different predictors may improve the overall prediction accuracy. This strategy is known as meta-strategy. In this project, the predictive results of five miRNA predictors: MiPred [35], MIReNA [38], miRPara [33], ProMiR [36], and triplet-SVM [31], were fed into an Artificial Neural Network (ANN) as shown in Fig 1 to produce a new prediction. The reasons for choosing these five predictors are as follows: (1) These five predictors are built using various machine learning techniques that are usually able to generate high-accuracy predictions; (2) These five predictors use different sequential, structural, and physicochemical features of RNA sequences as input, and are therefore able to ensure the divergence of true predictions on the same dataset; and (3) These five predictors have standalone versions. Since meta-predictor is a combination of individual predictors and there are in total five individual predictors, any number of individual predictors out of five can be used to compose a meta-predictor. Therefore, the total number of different types of combinations or the total number of different meta-predictors is:

C(5,2)+C(5,3)+C(5,4)+C(5,5)=26

### Artificial neural networks

As shown in Fig 1, the ANN has three layers: input layer, hidden layer, and output layer. The number of nodes in the input layer is the same as the number of individual predictors composed in the meta-predictor. In other words, the number of input nodes is two when two individual predictors were used to make the meta-predictor. When all the five individual predictors were used in the meta-predictor, the number of input nodes is five. The number of nodes in the hidden layer is determined by error and try. Multiple numbers of hidden nodes ranging from five to sixty were tested in the study. The final optimized meta-predictor mirMeta has 20 hidden nodes. The output layer has only one node. Logistic function was used as the activation function in all the hidden nodes and output node. The final output of ANN is a real number between 0 and 1. Output values larger than or equal to 0.5 were used to represent positive predictions, while output values smaller than 0.5 were assigned to negative predictions.

### Preprocessing input features by non-linear transformation

**Fig 2:**
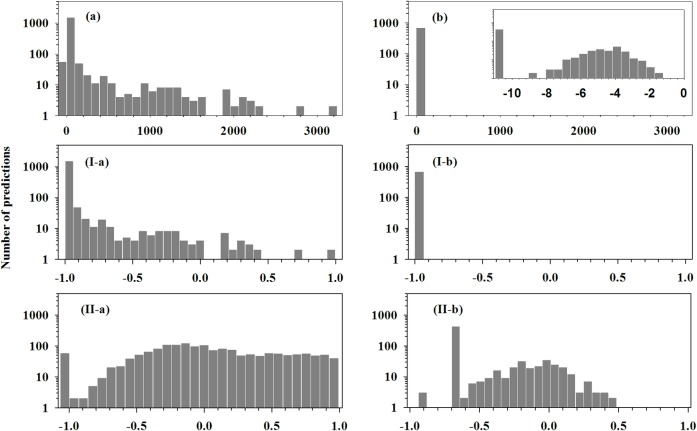
Non-linear transformations change the distribution of ProMiR prediction scores of all the samples in the D1679 dataset. The upper panels show the distribution of raw prediction scores of ProMiR for positive samples (a) and negative samples (b). The inset in (b) is the distribution of scores for negative samples when x-axis is scaled using logarithm. The intermediate panels present the distribution of prediction scores after preprocess-I transformation for positive samples (I-a) and negative samples (I-b). The lower panels are scores after preprocess-II transformation for positive samples (II-a) and negative samples (II-b).

The five individual predictors have two different types of output: binary output and real value output. MiReNA and triplet-SVM have binary outputs, while MiPred, miRPara, and ProMiR have numerical outputs. The ranges of the numerical outputs of these three predictors differ significantly as shown in Table 1. Clearly, all the outputs need to be numericalized and normalized before being fed into ANN. This process is called **preprocess-I** and includes the following steps: (1) transform all the binary outputs into 1 (positive prediction) or -1 (negative prediction); (2) normalize the outputs of each real-value predictor into the range (-1, 1). In our previous studies, non-linear transformation of input feature has been demonstrated to improve the prediction accuracy significantly [53]. Therefore, a specific transformation called **preprocess-II** was designed in the following way by focusing on shuffling the distribution of ProMiR predictions: (1) transform the outputs of the other four individual predictors except ProMiR using preprocess-I; (2) transform ProMiR predictions using the following procedure: (a) assign “-30” to all predictions labeled by “NA”; assign “-25” to all predictions with a numerical value of 0. These numbers were chosen by error and try; (b) calculate the logarithm of all positive numbers; (c) normalize the data into the range (-1,1). After taking this transformation, the distribution of outputs of ProMiR prediction was shifted significantly as shown in Fig 2. After using preprocess-II transformation, all the inputs can be further processed using the eigenvector matrix obtained from Principal Component Analysis (PCA) of the input data. This procedure is called **preprocess-III** and is described as follows: Assume there are N samples in the dataset and the meta-predictor comprises of M individual predictors (N>M, as we would expect in almost all the cases). The outputs of N samples from M predictors make an N*M output matrix. The eigenvector matrix E associated with this N*M output matrix has M*M dimensions. Each sample in the training dataset has M predictive results Y = (y_1_, y_2_,…y_M_), with each result from an individual predictor. For each sample, the predictive results Y can then be transformed into Y’ = (y_1_’, y_2_’,…y_M_’) using Y’ = Y*E, where E is the eigenvector matrix. Afterwards, y_1_’, y_2_’,…y_M_’ will be fed into ANN to train the predictor or to make meta-prediction.

**Table 1:** Possible outputs of five individual predictors.

	MiPred	MIReNA	miRPara	ProMiR	TripletSVM
Positive Prediction	50 ~ 91	Yes	0.8 ~ 1	0.017 ~ 3240	1
Negative Prediction	0	No	0	10e^-10^ to ~10e^-2^, 0, NA	NA

### Performance evaluation

While individual predictors have either numerical or binary outputs, the meta-predictor has only numerical output. Therefore, to ensure the consistency of comparison of prediction accuracy, the following four measures were used to evaluate the performance of predictors:

Sensitivity(SENS)=TP/(TP+FN)

Specificity(SPEC)=TN/(TN+FP)

Accuracy(ACC)=(TP+TN)/(TP+FP+FN+TN)

$$Matthews\;Correlation\;Coefficient\;(MCC)=\left(TP\times TN-FP\times FN\right)/\sqrt{\left(TP+FP)\times (TP+FN)\times (TN+FP)\times (TN+FN\right)}$$

In above equations, TP, FP, TN, and FN stand for true positive, false positive, true negative, and false negative rates of a predictor in a dataset, respectively. When optimizing meta-predictors, ACC was monitored to train ANN. However, when comparing the performance of different predictors, all four measures were used.

### Datasets

An existing dataset of 331 samples that was used by multiple miRNA predictors, such as triplet-SVM [31], MiPred [35], and ProMiR [36], was also used in this project. This dataset contains 163 non-redundant miRNA precursors obtained from an earlier version of miRBase [7] and 168 non-redundant hairpin-like pseudo-miRNA sequences extracted from human CDS regions. This dataset was named as D163 dataset in this study. The purpose of using the D163 dataset is to provide a common benchmark on which the newly developed meta-predictor can be compared with other individual predictors.

Another large dataset was developed for this project using the most recent MiRBase [7] and Rfam [57]. The current version of miRBase is the most prevailing and most comprehensive database of miRNAs, containing 1881 human miRNA precursors. After removing all the 163 positive samples in the D163 dataset, all the other 1718 precursors were used as positive samples in the large dataset. Another 2000 step-loop containing sequences with sequence identity less than 90% were selected randomly from Rfam to compose the negative samples. The RNA secondary structures of these samples were predicted using RNAfold [58]. All the samples in the dataset were predicted using five individual predictors. Samples with the same predictions from all five predictors were treated as “duplicates”. In this case, only one sample in each group of duplicates was selected randomly to compose the dataset. Finally, the dataset has 1679 real miRNAs and 674 pseudo miRNAs. This dataset was referred to as D1679 dataset.

### Validation

The accuracy of meta-predictor was systematically tested using two validation strategies: multi-fold cross-validation and validation on independent dataset. (1) Multi-fold cross-validation. When using multi-fold cross-validation, the dataset was divided randomly into multiple subsets, with roughly the same numbers of both positive and negative samples in each subset. Then, one subset was taken as the validation subset, another subset was selected as the test subset to prevent overfitting, and the rest subset(s) were used as training subset to optimize the predictor. At the end of each epoch during which all the samples in the training subset were used to optimize the predictor, the ACC value on the test subset was calculated and monitored. When the ACC value on the test subset stopped increasing for 100 epochs, the value of ACC on the validation subset was calculated as the validated accuracy of the meta-predictor. This process is repeated multiple times until every subset has been used as a validation subset. Finally, all the ACC values in all validation subsets were averaged to get the validated ACC under multi-fold cross validation. The number of subsets or the number of folds used in multi-fold cross-validation is determined by the size of the dataset. The D163 dataset was divided into three subsets (three-fold cross-validation), while the D1679 dataset was split into five subsets (five-fold cross-validation). The D1679 dataset was also split into three subsets to test the difference between three-fold cross validation and five-fold cross validation. As expected, the difference is negligible. (2) Validation on independent dataset. Since two independent datasets (D163 and D1679) were used in this project, predictors trained in one dataset were also validated using the other dataset as an independent test dataset.

### Final meta-predictor

The final version of meta-predictor mirMeta is a five-component predictor. The outputs of five individual predictors are processed using preprocess-III, which includes numericalization, normalization, distribution-shift of ProMiR predictions, and PCA transformation of all five individual predictions. The preprocessed data are then fed into an ANN, which has five input node, twenty hidden node, and one output node. The final output of mirMeta is a real number between 0 and 1. Values larger than 0.5 represent positive predictions and values less than 0.5 represent negative predictions.

## Results

### Performance of individual predictors in various datasets

Table 2 shows four different types of prediction accuracy (SENS, SPEC, ACC, and MCC) of five individual predictors on both D163 and D1679 datasets. In the D163 dataset, ProMiR achieved the best overall performance with ACC of 87.9% and MCC of 0.78. The other four predictors have similar overall performance, with ACCs at around 70% and MCCs at about 0.5 or slightly less. When comparing the values of SENS and SPEC, MiPred has the highest SENS of 99.4% but the lowest SPEC of 42.9%, ProMiR has the highest SPEC of 99.4% but the second lowest SENS of 76.1%. The other three predictors also show the similar discrepancy between the values of SENS and SPEC. MiRPara and triplet-SVM have high SENS of 91% and low SPEC of 52%, MiReNA has low SENS of 47% but high SPEC of 96%. In the D1679 dataset, MiPred becomes the top predictor with the highest ACC of 86.0% and the highest MCC of 0.65. These two values present significant increment of 16% and 0.14 compared to the values of the same predictor in the D163 dataset. The ACC and MCC values of MiReNA, MiRPara, and ProMiR in the D1679 dataset decrease significantly, compared to their values in the D163 dataset. Triplet-SVM gets similar ACC value but slightly decreased MCC value in the D1679 dataset compared to its values in the D163 dataset. In terms of SENS and SPEC, MiPred still gets the highest SENS, although the value decreases by ~8% to 91.6% compared to that in the D163 dataset. ProMiR has the highest SPEC of 99.1%, which is comparable to that in the D163 dataset. The other three predictors also show significantly decreased accuracies on either one or both of SENS and SPEC values.

**Table 2:** Prediction accuracies of five individual predictors in the D163 and D1679 datasets.

	D163				D1679			
	SENS	SPEC	ACC	MCC	SENS	SPEC	ACC	MCC
MiPred	99.4%	42.9%	70.7%	0.51	91.6%	72.0%	86.0%	0.65
MIReNA	47.2%	96.4%	72.2%	0.50	46.7%	69.3%	53.2%	0.15
MiRPara	91.4%	51.8%	71.3%	0.47	73.6%	38.6%	63.6%	0.12
ProMiR	76.1%	99.4%	87.9%	0.78	49.4%	99.1%	63.7%	0.46
Triplet-SVM	91.4%	51.8%	71.3%	0.47	84.8%	49.7%	74.8%	0.36

Clearly, the prediction accuracies of five individual predictors are dataset dependent. To further examine the performance of predictors, the true predictions of five individual predictors for both positive and negative samples was analyzed in both D163 dataset (Tables 3 and 4) and D1679 dataset (Tables 5 and 6). The numbers in each of the anti-diagonal cells in the tables represent the number of true predictions (above the slash) and the number of total samples (below the slash). Clearly, MiPred, MiRPara, and triplet-SVM have higher ratios of true prediction in positive samples, while MiReNA and ProMiR often have higher ratios of true prediction in negative samples. In all the non-anti-diagonal cells, the numbers above the slash specify the amount of true predictions overlapped between two predictors, while the numbers below the slash indicate the total number of non-redundant true predictions coming from two predictors. The number of overlapped true predictions indicates the similarity of two predictors, while the number of total true prediction from two predictors implies the coverage of true predictions of these two predictors in the dataset. For all the positive samples in the D163 dataset, triplet-SVM has the higher similarity with most of the other predictors. The next one that is similar to all other predictors is MiRPara, followed by MiPred and ProMiR. MiReNA has the least similarity with all other predictors. For negative samples in the D163 dataset, only MiReNA and ProMiR present high similarity to each other. In terms of coverage, all the binary combinations of five individual predictors are able to achieve high coverage for both positive and negative samples in the D163 dataset. In the D1679 dataset, MiPred, Triplet-SVM, and MiRPara have higher similarity with other predictors than ProMiR and MIReNA for positive samples. For negative samples in the D1679 dataset, only three pairs of predictors, which are MiPred and MiReNA, MiReNA and MiRPara, MiReNA and ProMiR, have over 50% of similarity. Therefore, MiReNA becomes the only predictor that has reasonably high similarity with all other predictors. In terms of coverage, MiPred has the largest coverage with any of the other predictors in both positive and negative samples in the D1679 dataset. The maximal coverage from two individual predictors for positive samples in the D1679 dataset is 1608, which comes from MiPred and MiRPara. The maximal coverage on negative samples in the same dataset is 664, which is obtained from MiPred and MiReNA. These two coverage values stand for 95.8% of SENS and 98.5% of SPEC, or in general 96.6% of ACC in the D1679 dataset. However, the highest SENS, SPEC, and ACC of five individual predictors in the D1679 dataset as shown in Table 2 are only 91.6%, 72.0%, and 86.0%, respectively. These numbers indicate that the combination of these individual predictors may improve prediction accuracy in the D1679 dataset.

**Table 3:** Comparison of true predictions between every two individual predictors for all the 163 positive samples in the D163 dataset.

	MiPred	MIReNA	MiRPara	ProMiR	Triplet-SVM
MiPred	(162/163)	77/162	149/162	77/163	149/162
MIReNA	---	(77/163)	65/161	60/141	71/155
MiRPara	---	---	(149/163)	115/158	137/161
ProMiR	---	---	---	(124/163)	118/155
TripSVM	---	---	---	---	(149/163)

In each of the diagonal cells, the number above the slash is the number of true positive (TP) of that predictor, while the number below the slash shows total number of samples. In each of the non-anti-diagonal cells, the number above the slash represents the number of overlapped TP predictions between two predictors, while the number below the slash is the total number of non-redundant TP prediction of two predictors.

**Table 4:** Comparison of true predictions between every two individual predictors for all the 168 negative samples in the D163 dataset.

	MiPred	MIReNA	MiRPara	ProMiR	Triplet-SVM
MiPred	(72/168)	70/164	45/141	72/168	68/158
MIReNA	---	(162/168)	87/162	161/168	83/166
MiRPara	---	---	(87/168)	87/168	35/139
ProMiR	---	---	---	(167/168)	86/168
TripSVM	---	---	---	---	(154/168)

In each of the diagonal cells, the number above the slash is the number of true negative (TN) of a predictor, while the number below the slash shows total number of samples. In each of the non-anti-diagonal cells, the number above the slash represents the number of overlapped TN predictions between two predictors, while the number below the slash is the total number of non-redundant TN predictions of two predictors.

**Table 5:** Comparison of true predictions between every two individual predictors for all the 1679 positive samples in the D1679 dataset.

	MiPred	MIReNA	MiRPara	ProMiR	Triplet-SVM
MiPred	(1538/1679)	748/1575	1166/1608	777/1591	1368/1594
MIReNA	---	(785/1679)	553/1468	494/1121	743/1466
MiRPara	---	---	(1236/1679)	625/1441	1127/1533
ProMiR	---	---	---	(830/1679)	782/1472
TripSVM	---	---	---	---	(1424/1679)

In each of the diagonal cells, the number above the slash is the number of true positive (TP) predictions of a predictor, while the number below the slash shows total number of samples. In each of the non-anti-diagonal cells, the number above the slash represents the number of overlapped TP predictions between two predictors, while the number below the slash is the total number of non-redundant TP predictions of two predictors.

**Table 6:** Comparison of true predictions between every two individual predictors for all the 674 negative samples in the D1679 dataset.

	MiPred	MIReNA	MiRPara	ProMiR	Triplet-SVM
MiPred	(485/674)	288/664	120/625	482/668	171/536
MIReNA	---	(467/674)	251/476	462/673	197/492
MiRPara	---	---	(260/674)	258/670	137/345
ProMiR	---	---	---	(668/674)	221/669
TripSVM	---	---	---	---	(222/674)

In each of the diagonal cells, the number above the slash is the number of true negative (TN) predictions of a predictor, while the number below the slash shows total number of samples. In each of the non-anti-diagonal cells, the number above the slash represents the number of overlapped TN predictions between two predictors, while the number below the slash is the total number of non-redundant TN predictions of two predictors.

### Integration of meta-strategy and simple numericalization transformation

**Fig 3:**
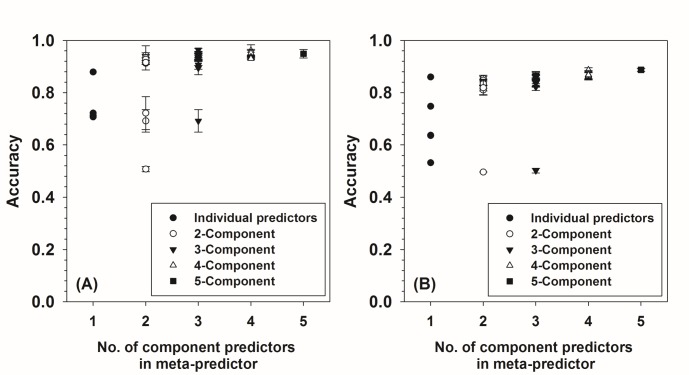
**Prediction accuracies of meta-predictors made from various combinations of five individual predictors in the (A) D163 and (B) D1679 datasets.** The input of ANN in the meta-predictor were preprocessed using preprocess-I transformation. X-axis shows the number of individual predictors in the meta-predictor, while y-axis shows the prediction accuracy (ACC) under three-fold cross validation (A) and five-fold cross validation (B). In the case that x equals to 1, y-axis shows the prediction accuracies of five individual predictors. The numbers of meta-predictors composed of 2, 3, 4, and 5 individual predictors are 10, 10, 5, and 1, respectively.

As outlined in Fig 1, meta-strategy was applied to build meta-predictors based on all 26 combinations of five individual predictors. Since these five individual predictors have both numerical and binary outputs, their outputs were transformed using preprocess-I transformation. The accuracies (ACCs) of these meta-predictors under multi-fold cross validation in both the D163 and the D1679 datasets were presented in Fig 3. As shown in this figure, the lowest prediction accuracy of meta-predictors is always higher than the lowest of the individual predictors, and the highest accuracy of meta-predictors is always higher (or at least not lower) than the highest of the individual predictors. In addition, adding more component predictors into a meta-predictor may generally improve the prediction performance of the meta-predictor. These observations strongly substantiate that the meta-strategy improves the prediction accuracy. By comparing Fig 3(A) with 3(B), it can also be seen that all the meta-predictors trained in the D1679 dataset have lower accuracies than the meta-predictors trained in the D163 dataset. The reason is that the accuracies of individual predictors are normally lower in the D1679 dataset than in the D163 dataset. The highest accuracy (ACC) of meta-predictors in the D163 dataset was achieved by a combination of four individual predictors including: MiPred, MIReNA, miRPara, and ProMiR. The accuracy of this meta-predictor is 96.1±2.2% under three-fold cross validation in the D163 dataset. This value is about 8% higher than the highest ACC value in the same dataset achieved by a single predictor, which is 87.9% by ProMiR (Table 2). This meta-predictor is called Meta-I-4S in the following discussion. Here, “I” stands for preprocess-I, “4” indicates there are four individual predictors in the meta-predictor, and “S” shows that the meta-predictor was trained in the D163 dataset. In the D1679 dataset, the highest accuracy was achieved by the meta-predictor composed of all five individual predictors. The accuracy is 86.6% under five-fold cross-validation in the D1679 dataset. This accuracy is slightly higher than the highest ACC value of individual predictors in the D1679 dataset, which is 86% by MiPred (Table 2). This meta-predictor is named after Meta-I-5L, where “I” stands for preprocess-I, “5” indicates the meta-predictor has five individual predictors, and “L” denotes that the predictor has been trained in the D1679 dataset. Three-fold cross validation was also applied to all the meta-predictors trained in the D1679 dataset. However, the differences from the results in the five-fold cross validation are neglectible.

In addition to the ACC values under multi-fold cross-validation, the other three types of accuracy for Meta-I-4S and Meta-I-5L under multi-fold cross-validation, as well as the accuracies of Meta-I-4S and Meta-I-5L in the independent dataset were tested and presented in Table 7. For Meta-I-4S, the accuracies under three-fold cross validation in the D163 dataset have been significantly improved over the accuracies of ProMiR, which is the top predictor in the D163 dataset. The values of SENS, SPEC, ACC, and MCC for Meta-I-4S are 93.3%, 98.8%, 96.1%, and 0.92, with increments of 17%, -1%, 8%, and 0.14 over the corresponding values of ProMiR. When comparing different types of accuracies of Meta-I-4S to the highest values of individual predictors that are shown in Table 2, Meta-I-4S still has considerable advantages. Although it is 6% behind MiPred in SENS and 1% lower than the SPEC of ProMiR, Meta-I-4S has 55% higher SPEC than MiPred and 17% higher SENS than ProMiR. In addition, Meta-I-4S is 8% and 25% higher in ACC than the values of ProMiR and MiPred, and has increments of 0.14 and 0.41 in MCC compared to that of ProMiR and MiPred. In terms of accuracies in independent dataset, the performance of Meta-I-4S in the D1679 dataset decreased considerably compared to the values under three-fold cross validation in the D163 dataset with decrements of 5%, 6%, 7%, and 0.16 for SENS, SPEC, ACC, and MCC, respectively. Even though, the accuracies of Meta-I-4S in the D1679 dataset are still in general higher than the accuracies of MiPred that has achieved the best prediction accuracy in the D1679 dataset, by -3%, 20%, 10%, and 0.27 for SENS, SPEC, ACC, and MCC, accordingly. For Meta-I-5L, the accuracies under five-fold cross validation in the D1679 dataset are 79%, 94%, 87%, and 0.74, for SENS, SPEC, ACC, and MCC, respectively. These values are lower than the accuracies of Meta-I-4S under three-fold cross validation in the D163 dataset. In addition, the accuracies of Meta-I-5L in D1679 under five-fold cross validation are also lower than those of Meta-I-4S in the D1679 dataset as an independent test. Nonetheless, the performance of Meta-I-5L in the independent test dataset D163 is comparable to the accuracies of Meta-I-4S under three-fold cross validation in the D163 dataset. In brief, although the performances of meta-predictors vary a lot, the meta-predictors achieve better balance between SENS and SPEC, improve the overall prediction accuracy, and reduce the dependence on dataset.

**Table 7:** Performance of meta-predictors using preprocess-I transformation under multi-fold cross validation and in independent dataset.

Predictor	Dataset	SENS	SPEC	ACC	MCC
Meta-I-4S	D163*	93.3 ± 2.8%	98.8 ± 1.6%	96.1 ± 2.2%	0.92 ± 0.04
	D1679**	88.1 ± 2.4%	92.9 ± 0.1%	89.5 ± 1.7%	0.76 ± 0.03
Meta-I-5L	D1679*	79.4 ± 4.4%	94.0 ± 1.3%	86.6 ± 1.6%	0.74 ± 0.03
	D163**	98.9 ± 0.4%	93.2 ± 3.3%	96.0 ± 1.4%	0.92 ± 0.03

(*) Meta-I-4S is composed of four individual predictors: MiPred, miReNA, MiRPara, and ProMiR. The predictor was optimized in the D163 dataset using three-fold cross validation; Meta-I-5L is composed of five individual predictors: MiPred, miReNA, MiRPara, ProMiR, and TripSVM. It was trained in the D1679 dataset using five-fold cross validation.

(**) The performance of these two predictors in independent dataset, which was D1679 for Meta-I-4S and D163 for Meta-I-5L, was averaged over three- or five-iterations of prediction that correspond to three- or five-fold cross validation. Errors were standard errors calculated from either three- or five-iterations of prediction.

### Integration of meta-strategy and distribution-shift transformation

To further improve the accuracies of meta-predictors, preprocess-II transformation was applied on the input of meta-predictors. This time, a meta-predictor composed of MiPred and ProMiR achieved the highest accuracy in the D163 dataset under three-fold cross validation, and another meta-predictor made of all five individual predictors achieved the highest accuracy under five-fold cross validation in the D1679 dataset. These two meta-predictors were called Meta-II-2S and Meta-II-5L, respectively. These two meta-predictors were also tested in independent dataset as well. The results for the accuracies of these two predictors under multi-fold cross validation and in independent dataset were shown in Table 8. When comparing the results of Table 8 to that in Table 7, it can be seen that the application of preprocess-II transformation has improved the accuracies of meta-predictors under multi-fold cross validation by at least 2–3%. The SENS, SPEC, ACC, and MCC values of Meta-II-2S in the D163 dataset under three-fold cross validation are 96%, 100%, 98%, and 0.96, with increments of 3%, 1%, 2%, and 0.04 over the accuries of Meta-I-4S in the same dataset under three-fold cross validation. However, in the independent dataset D1679, the performance of Meta-II-2S decreases obviously compared to the performance of Meta-I-4S in the D1679 dataset. The decrements are 14%, -2%, 10%, and 0.14 for SENS, SPEC, ACC, and MCC, accordingly. In terms of Meta-II-5L, the SENS, SPEC, ACC, and MCC values under five-fold cross-validation in the D1679 dataset are increased by 3%, 1%, 2%, and 0.04, compared to the values of Meta-I-5L under five-fold cross validation in the same dataset. When using the D163 dataset as an independent dataset, the performance of Meta-II-5L is about 1~2% lower than Meta-I-5L in the D163 dataset. By comparing to the accuracies of individual predictors in Table 2, the advantages of the combination of meta-strategy and preprocess-II transformation becomes obvious. Meta-II-2S exceeds all the individual predictors in the D163 dataset in almost all types of accuracy. The only exception is the SENS of Meta-II-2S, which is 96% and is 3% lower than that of MiPred. However, the other types of accuracy of MiPred are much lower than those of Meta-II-2S. In the D1679 dataset when performing the independent test, Meta-II-2S is still comparable to MiPred, the best individual predictor that has achieved the highest prediction accuracy in the D1679 dataset. For Meta-II-5L, the advantages over individual predictors are more evident. The ACC and MCC values of Meta-II-5L under five-fold cross validation in the D1679 dataset are 88.7% and 0.78, which are about 2% and 0.13 higher than those of MiPred in the same dataset. The SENS of Meta-II-5L in the D1679 dataset under five-fold cross validation is 9% lower than that of MiPred, but the SPEC of Meta-II-5L is 23% higher than that of MiPred. In the D163 dataset during independent test, the accuracies of Meta-II-5L are systematically higher than the numbers of individual predictors in the same dataset. The SENS of Meta-II-5L is the same as MiPred, and the SPEC, ACC, and MCC values of Meta-II-5L are higher than those of MiPred by 47%, 24%, and 0.39 in the D163 dataset. When comparing with ProMiR in the D163 dataset, Meta-II-5L’s SPEC is lower by 9%, but the SENS, ACC, and MCC values of Meta-II-5L are higher by 23%, 7%, and 0.12, respectively. Therefore, a large training dataset is also helpful in improving the prediction accuracy of meta-predictors.

**Table 8:** Performance of meta-predictors under multi-fold cross validation and in independent dataset under preprocess-II transformation strategy.

Predictor	Dataset	SENS	SPEC	ACC	MCC
Meta-II-2S	D163*	96.3 ± 1.2%	1.0 ± 0.0%	98.2 ± 0.6%	0.96 ± 0.01
	D1679**	73.8 ± 4.5%	94.6 ± 1.0%	79.8 ± 2.9%	0.62 ± 0.04
Meta-II-5L	D1679*	82.5 ± 1.9%	95.0 ± 1.7%	88.7 ± 0.6%	0.78 ± 0.01
	D163**	99.4 ± 0.0%	90.5 ± 0.6%	94.9 ± 0.3%	0.90 ± 0.01

(*) Meta-II-2S is composed of MiPred and ProMiR. The predictor was optimized in the D163 dataset using three-fold cross validation; Meta-II-5L is composed of all the five individual predictors including: MiPred, miReNA, MiRPara, ProMiR, and TripSVM. It was trained in the D1679 dataset using five-fold cross validation.

(**) The performance of these two predictors in independent dataset, which was D1679 for Meta-II-2S and D163 for Meta-II-5L, was averaged over three- or five-iterations of prediction that correspond to three- or five-fold cross validation. Errors were standard errors calculated from either three- or five-iterations of prediction.

### Integration of meta-strategy, distribution-shift transformation, and PCA transformation

Preprocess-III transformation was then incorporated in the meta-predictors. The selected meta-predictors with the highest accuracies under multi-fold cross-validation in the D163 and D1679 datasets are Meta-III-5S and Meta-III-5L, respectively. Their accuracies were compared in Table 9. For Meta-III-5S, the accuracies under three-fold cross validation in the D163 dataset are slightly lower than those of Meta-II-2S in the D163 dataset, but are essentially the same as those of Meta-I-4S in the D163 dataset (Table 7). In addition, the performance of Meta-III-5S in the D1679 dataset as an independent test is slightly lower than those of Meta-I-4S in the D1679 dataset, and is generally better than that of Meta-II-2S in the D1679 dataset. For Meta-III-5L, the SENS, SPEC, ACC, and MCC values under five-fold cross validation in the D1679 dataset are 89%, 98%, 93%, and 0.87, which are improved by 7%, 2%, 5%, and 0.09 compared to the accuracies of Meta-II-5L in the D1679 dataset (Table 8). In the independent test dataset D163, the accuracies of Meta-III-5L are the same as those of Meta-II-5L in the D163 dataset and are comparable to those of Meta-I-4S in the D163 dataset. When comparing to individual predictors, both Meta-III-5S and Meta-III-5L demonstrate significantly improved performance compared to individual predictors. When comparing Meta-III-5S to Meta-III-5L, it is clear that the ACC values of Meta-III-5L are higher and more consistent in different datasets than those of Meta-III-5S.

**Table 9:** Performance of meta-predictors under multi-fold cross validation and in independent dataset under preprocess-III transformation strategy.

Predictor	Dataset	SENS	SPEC	ACC	MCC
Meta-III-5S	D163*	93.1 ± 2.1%	1.0 ± 0.0%	96.6 ± 1.0%	0.93 ± 0.02
	D1679**	91.4 ± 0.1%	83.7 ± 0.1%	89.2 ± 0.1%	0.74 ± 0.00
Meta-III-5L	D1679*	89.1 ± 3.2%	97.7 ± 0.4%	93.4 ± 1.7%	0.87 ± 0.03
	D163**	99.4 ± 0.0%	90.1 ± 0.1%	94.7 ± 0.7%	0.90 ± 0.01

(*) Meta-III-5S and Meta-III-5L are composed of five individual predictors, which are: MiPred, miReNA, MiRPara, ProMiR, and TripSVM. Meta-III-5S was optimized in the D163 dataset using three-fold cross validation, while Meta-III-5L was trained in the D1679 dataset using five-fold cross validation.

(**) The performance of these two predictors in independent dataset, which was D1679 for Meta-III-5S and D163 for Meta-III-5L, was averaged over three- or five-iterations of prediction that correspond to three- or five-fold cross validation. Errors were standard errors calculated from either three- or five-iterations of prediction. Meta-III-5L has the best overall performance, and is therefore used as the final meta-predictor mirMeta.

### Comparison with existing predictor

The infrastructure of Meta-III-5L was used to build the final meta-predictor mirMeta. The performance of mirMeta was compared with HeteroMirPred, which is another well-designed meta-predictor and the only existing meta-predictor for miRNA to the best of our knowledge. The biggest differences between mirMeta and HeteroMirPred in terms of overall strategy are: (1) mirMeta used ANN to combine the predictive results of individual predictors. As a comparison, HeteroMirPred used majority-voting; (2) the predictive results of individual predictors were preprocessed by various non-linear transformations before being fed into ANN. In HeteroMirPred, the results of individual predictors were used directly for the next step. Due to these differences, mirMeta outperformed HeteroMirPred in multiple aspects as shown in Table 10. In small dataset D163, the SPEC, ACC, and MCC values of mirMeta are lower than those of HeteroMirPred by small numbers, although the SENS value of mirMeta is still higher than that of HeteroMirPred. In large dataset D1679, the performance of mirMeta is clearly much better that that of HeteroMirPred.

**Table 10:** Comparison between mirMeta and HetroMirPred.

Predictor	Dataset	SENS	SPEC	ACC	MCC
mirMeta	D1679^(a)^	89.1 ± 3.2%	97.7 ± 0.4%	93.4 ± 1.7%	0.87 ± 0.03
	D163^(b)^	99.4%	90.1%	94.7%	0.90
HeteroMirPred	D1679^(b)^	78.1%	52.5%	69.6%	0.29
	D163^(b)^	98.7%	96.6%	97.6%	0.95

^(a)^ The accuracies of mirMeta in D1679 were obtained from five-fold cross-validation. The values are the same as those in Table 9.

^(b)^ The accuracy were calculated using D163 dataset as an independent dataset.

## Discussion

With the fast development of RNAseq techniques in recent years, identifying functional non-coding RNAs becomes more and more critical for biological and biomedical studies. MiRNAs, being the largest category of gene expression regulators, have been given more and more attentions. In this field, although more than 2,000 miRNAs have been validated in human, it is still not clear if or not novel human miRNAs can be discovered. From a mechanistic point of view, the biogenesis of miRNAs is determined by the sequential and structural features of the precursor miRNAs. Therefore, SNPs and other mutations on the precursor miRNAs may influence the production of miRNAs. Since SNPs and mutations are prevalent and may vary among individuals, tissues, and diseases, identifying novel miRNAs becomes possible and critical. From the systematical point of view, identifying the complete set of miRNAs that are specific to a certain condition is even more critical. However, state of the art miRNA predictors may not be able to perform well for this specific objective due to issues in their training process and in their performances.

Computational predictors are optimized using specific training datasets. Therefore, the performances of the same predictor in different datasets may be different as shown in Table 2. This is the dataset dependence of predictors. Many state of the art miRNA predictors were optimized using small training dataset of several hundreds of samples. Therefore, the performance of these predictors in large dataset becomes a concern. In addition, the computational predictors are often designed using different computational strategies and input features. Consequently, different predictors may have very different prediction accuracies on the same dataset. Moreover, these predictors may be optimized for high sensitivity or high specificity as shown in Table 2, in which the values of SENS, SPEC, ACC, and MCC for MiPred, MiReNA, MiRPara, ProMiR, and Triplet-SVM in the D163 and D1679 datasets change significantly. These issues are critical for not only miRNA predictors, but also all other computational tools based on supervised training. Therefore, how to improve the robustness of prediction accuracy of predictors in different datasets, to improve the balance between SENS and SPEC, and to further improve the prediction accuracy become very critical in the field of computational biology.

By analyzing the true predictions in both positive and negative samples between every two individual miRNA predictors, it was found that some pairs of predictors have high overlap of true predictions in the same set of samples. The high level of overlap between two predictors suggests that these two predictors are similar to each other in terms of the output of prediction. When comparing total number of non-redundant true predictions generated by each pair of predictors, some pairs of individual predictors showed higher numbers of non-redundant true predictions in the positive samples, negative samples, or both of them. The total number of non-redundant true predictions from a pair of predictors provides a measure of the coverage of true predictions in a specific dataset. This coverage specifies theoretically the highest prediction accuracy that can be achieved by using both of the predictors simultaneously. As shown by the data in Tables 3–6, the highest values of coverage in different datasets are much higher than the highest prediction accuracy of individual predictors in that dataset. Therefore, combining different predictors to get better prediction becomes a natural choice. This strategy has been well known as meta-strategy, and the predictor developed using this strategy is called meta-predictor. In this project, ANN was used to integrate different individual predictors, the parameters of ANN were optimized in large datasets, and the performances of meta-predictors were evaluated using multi-fold cross validation and using independent dataset. The results have demonstrated that meta-predictors exceed individual predictors in the following three aspects: (1) Meta-predictor is able to achieve higher prediction accuracy; (2) Meta-predictor has much better balanced values between SENS and SPEC; (3) The accuracy of meta-predictor is more dataset-independent compared to individual predictors; and (4) Meta-predictor trained in large dataset is normally more dataset-independent and therefore is able to provide more reliable predictions.

The meta-predictors take the output of individual predictors as input. The predictive results of different individual predictors in the same set of samples may have different distributions. As being observed in previous projects, the distribution may influence the prediction accuracy of meta-predictor. To evaluate the influence, we designed three different transformations named after preprocess-I, II, and III. Basically, preprocess-I is a binary-numerical transformation, preprocess-II incorporates nonlinear transformation to change the distribution of outputs of individual predictors, preprocess-III integrates principal component analysis into the transformation based on the outcome of preprocess-II. These transformations have significant influence on the prediction accuracy of meta-predictors. As shown by the data in Tables 7–9, the combination of meta-strategy and preprocess-I barely improves the prediction accuracy in terms of ACC value. However, replacing preprocess-I by preprocess-II in the meta-predictor is able to improve the prediction accuracy by ~3%. Furthermore, integrating meta-strategy and preprocess-III increases the prediction accuracy to 93%, which represents an increment of 7% compared to the highest accuracy of individual predictors.
